# Correlation Between Oral Lichen Planus and Thyroid Disease in China: A Case–Control Study

**DOI:** 10.3389/fendo.2018.00330

**Published:** 2018-06-18

**Authors:** Tingting Zhou, Dan Li, Qianming Chen, Hong Hua, Chunlei Li

**Affiliations:** ^1^Department of Oral Medicine, Peking University School and Hospital of Stomatology, Beijing, China; ^2^Department of Oral Medicine, West China School of Stomatology, SiChuan University, Chengdu, China

**Keywords:** oral lichen planus, oral lichenoid lesion, Hashimoto’s thyroiditis, hypothyroidism, thyroid nodule, molecular mimicry

## Abstract

**Background:**

A possible relationship between oral lichen planus (OLP) and thyroid disease has received attention in recent years.

**Objective:**

This study aimed to evaluate the correlation between OLP and thyroid diseases in Chinese ethnic patients.

**Methods:**

192 OLP patients, 123 patients with oral lichenoid lesions (OLLs), and 162 controls were recruited in this case–control study. All participants received screening for thyroid function and underwent ultrasound. Sex and age of the patients in the three groups were matched. The prevalence of thyroid diseases in the subjects was analyzed. Using logistic regression, the odds ratio (OR) with 95% confidence intervals was appraised for associations between OLP, OLL, and different types of thyroid diseases [Hashimoto’s thyroiditis (HT), hypothyroidism, and thyroid nodule].

**Results:**

The prevalence of thyroid diseases in the OLP group (72.4%) and OLL group (68.3%) was higher than the control group (49.4%) with statistical significance. The OR of HT was 3.16 (1.87–5.33) for OLP, 2.09 (1.18–3.70) for OLL, while the OR of thyroid nodule was 2.31 (1.30–4.09) for OLP.

**Conclusion:**

Our study suggested a close relationship between OLP/OLL and HT and thyroid nodule in a Chinese population. The possible mechanism behind this association warrants further investigation.

## Introduction

Oral lichen planus (OLP) is a chronic autoimmune oral mucosa disease with a prevalence of 1–2% in the general population, which most commonly affects middle-aged and elderly female (1, 2). It has been defined as a potentially malignant disorder of the oral cavity (OPMD) (3). According to the World Health Organization (WHO), the estimated malignant transformation rate ranges from 1.09 to 1.14% (4). Despite extensive studies, the etiology and pathogenesis of OLP remains unknown. Various factors such as genetic predisposition, psychological factors, and infectious precipitants may be involved in the development of OLP (5, 6). Moreover, some systemic diseases such as hypertension, diabetes, and thyroid disorders may increase the risk of OLP (7–9).

Oral lichenoid lesions (OLLs) share some common clinical and histological features with OLP, which are often indistinguishable in their manifestation. The etiological factors for OLL have been identified in clinic and are commonly related to specific drugs, dental material, or graft-versus-host disease (10). But there were no special inducing factors mentioned above in most OLL patients.

Thyroid disease is a common endocrine systemic disease and causes serious public health problems. The prevalence of goiter is near 80% in severe iodine deficiency areas. The neonatal morbidity of congenital hypothyroidism can be 1/3,500–4,000 which is also the primary risk of mental retardation (11). Other thyroid disorders such as hyperthyroidism, thyroid cancer, and autoimmune thyroiditis have a diverse prevalence ranging from 0.5 to 1% in different countries (12). In China, over two billion people suffer from thyroid diseases, where the prevalence of hypothyroidism and hyperthyroidism has been estimated to be 6.5 and 1.3%, respectively, according to an epidemiological survey conducted in 2013. The prevalence of thyroid nodules was 12.8%, but this may be underestimated due to low sensitivity of the ultrasonographic equipment (13). In 2016, a study on the prevalence of thyroid diseases in China was conducted by Chinese Society of Endocrinology revealed that more than 40% of the population have thyroid diseases (14). An increasing number of studies have investigated the possible relationship between thyroid disease and OLP in other populations (15–17). In our clinical practice, we also observed that many OLP and OLL patients often have a diagnosis of thyroid diseases. However, there is no robust evidence regarding this association in the Chinese ethnic population in the literature to date.

Therefore, we conducted this case–control study to assess the possible relationship between OLP, OLL, and thyroid diseases in Chinese ethnic population.

## Materials and Methods

### Ethical Approval

This study was approved by the Peking University Institutional Review Board, China [PKUSS IRB _AF 01 _v2.0 (2014-10-01)], and all methods were performed in accordance with the relevant guidelines and regulations. Each subject agreed and provided written informed consent before the study.

### Patients

There were all together 477 patients referred to the Department of Oral Medicine, Peking University School and Hospital of Stomatology, China from October 10th, 2014 to April 30th, 2017, were recruited to the case–control study, including OLP patients, OLL patients, and controls with other diseases including burning mouth syndrome, recurrent aphthous ulcers, and geographic tongue. The controls also included some healthy person without any oral mucosal diseases. All participants were matched for age and sex.

#### Inclusion Criteria

The subjects were recruited according to the following criteria. OLP and OLL patients who were diagnosed clinically and histologically according to WHO diagnostic criteria (18). Briefly for the diagnosis of OLP, the clinical criteria included the presence of bilateral, more or less symmetric lesions; the lesions could be lacelike network of gray–white lines, plaque, atrophic, bullous, or erosive type. The histological criteria included the presence of well-defined band-like zone of cellular infiltration confined to the superficial part of the connective tissue, sign of liquefaction degeneration in the basal cell layer and the absence of epithelial dysplasia. Patients who were clinically or/and histologically compatible with OLP were diagnosed as OLL.

According to the guideline of diagnosis and treatment of thyroid diseases in China (19), the criteria for Hashimoto’s thyroiditis (HT) included the positive detection of thyroid peroxidase antibody (TPOAb) and thyroglobulin (TGAb), with or without local and systemic manifestations including dysphonia, dyspnea, and constipation (20). For hypothyroidism, it included the higher value of thyroid-stimulating hormone (TSH), the lower value of total thyroxine (TT4) and free thyroxine (FT4). For thyroid nodules, it included one or more lump in the thyroid gland detected by B ultrasound. For hyperthyroidism, it included the lower value of TSH, the higher value of TT4, FT4, total triiodothyronine (TT3), and free triiodothyronine (FT3), with or without clinical symptoms and signs of clinical hypermetabolism, such as increased heart rate.

#### Exclusion Criteria

Patients under 18 years old or who were pregnant; patients with a history of action capability restricting or life-threatening systemic diseases like uremia, or other autoimmune diseases which seriously affects the quality of life, such as psoriasis, vitiligo, Behcet disease, or bullous diseases.

### Data Collection

The demographic and clinical information from the patients was recorded including age, gender, site of oral lesion, medication history, and general condition.

All subjects were instructed to undergo a thyroid examination including thyroid function (TT3, TT4, FT3, FT4, and TSH), thyroid related antibodies (TPOAb and TGAb). These indicators were detected by chemiluminescence immunoassay (CLIA). Patients also received a B ultrasound of the thyroid gland (19, 21).

Results of the thyroid examination were determined by two senior endocrinologists of Peking University First Hospital to confirm the diagnosis of HT, hypothyroidism, thyroid nodule, and hyperthyroidism accordingly. The thyroid diseases of the subjects were newly diagnosed.

### Statistical Analysis

IBM SPSS statistical software (version 19.0, IBM Crop, Armonk, New York, NY, USA) was used for data recording and analyzing.

The prevalence of thyroid diseases in the three groups was calculated. The differences were evaluated by the chi-square test. Furthermore, we calculated the odds ratio (OR) with 95% confidence intervals (CI) for associations between OLP, OLL, and different types of thyroid diseases (HT, hypothyroidism, and thyroid nodule) using logistic regression adjusted for matched age and sex. The significance level was set as *P* < 0.05.

## Results

### Demographic Information

192 OLP patients (24.45% male and 75.5% female; mean age: 49.53 ± 9.93 years), 123 OLL patients (23.6% male and 76.4% female, mean age: 50.46 ± 9.38 years), and 162 control subjects (19.75% male and 80.25% female; mean age: 50.96 ± 8.96 years) were assessed in our study. The subjects were appropriately age and sex matched. The information of OLP/OLL and control group patients is shown in Table 1.

**Table 1:** Demographics information of the oral lichen planus (OLP)/oral lichenoid lesion (OLL) and control groups.

	OLP (*n* = 192)	OLL (*n* = 123)	Control (*n* = 162)
**Age** (years)	49.53 ± 9.93	50.46 ± 9.38	50.96 ± 8.96
**Gender**			
Male (%)	47 (24.48)	29 (23.58)	32 (19.75)
Female (%)	145 (75.52)	94 (76.42)	130 (80.25)
Smoking history (%)	10 (5.21)	7 (5.69)	8 (4.94)
Daily medications (%)	32 (16.67)	20 (16.26)	23 (14.20)
**Systemic diseases**			
Hypertension (%)	29 (15.10)	22 (17.89)	24 (14.81)
Diabetes (%)	6 (3.13)	5 (4.07)	6 (3.70)
Hepatitis (%)	4 (2.08)	3 (2.44)	3 (1.85)

### Clinical Characteristics

About 2/3 OLP and OLL patients are non-erosive in clinical type, and the condition of lesion location is shown in Table 2.

**Table 2:** Clinical characteristics of oral lichen planus (OLP)/oral lichenoid lesion (OLL) group patients.

	OLP, *n*(%)	OLL, *n*(%)
**Clinical type**		
Erosive	50 (26.00)	41 (33.30)
Non-erosive	142 (74.00)	82 (66.70)
**Site of lesion**
Buccal only	43 (22.40)	38 (30.90)
Buccal and gingival	23 (12.00)	5 (4.00)
Buccal and lingual	53 (27.60)	37 (30.10)
Buccal, gingival and lingual	12 (6.30)	12 (9.80)
Lingual only	16 (8.30)	5 (4.00)
Buccal and lip	19 (9.90)	7 (5.70)
Other^a^	26 (13.50)	19 (15.20)
Coexistent with cutaneous LP	13 (6.77)	6 (4.88)

*^a^Lip, gingival, palate, floor of mouth only, or multilocalized lesions*.

### OLP/OLL and Thyroid Diseases

The prevalence rate of thyroid disease in OLP and OLL groups was remarkably higher than control group (Table 3). In particular, HT was significantly connected to OLP and OLL; moreover, thyroid nodules were positively associated with OLP but not OLL. Hypothyroidism showed no significant association with OLP/OLL (Table 4). Thyroid hormones and autoantibodies level of OLP/OLL and control are shown in Table 5.

**Table 3:** Prevalence of thyroid diseases in oral lichen planus (OLP), oral lichenoid lesion (OLL), and control group.

	OLP	OLL	Control	*P* value
Thyroid diseases, *n* (%)	139 (72.40)	84 (68.30)	80 (49.40)	<0.0001*

*The P value is the result of a comparison between OLP and Control group, as well as OLL and Control group.

**Table 4:** Thyroid diseases in oral lichen planus (OLP) and oral lichenoid lesion (OLL) patients and control group by risk factor analysis.

Group	Control	OLP			OLL		
	*n*	*n*	*P* value	OR [95% confidence intervals (CI)]	*n*	*P* value	OR (95% CI)
Hashimoto thyroiditis	16	40	0.000	3.160 (1.874, 5.328)	23	0.011	2.090 (1.181, 3.698)
Hypothyroidism	8	10	0.225	1.858 (0.683, 5.059)	6	0.565	1.389 (0.453, 4.256)
Thyroid nodule	33	47	0.004	2.306 (1.299, 4.094)	28	0.156	1.570 (0.842, 2.926)
Hyperthyroidism	1	3	0.402	–	1	0.845	–
Multiple^a^	22	39	–	–	26	–	–
None	82	53	–	–	39	–	–

*^a^Coexistence of multiple thyroid diseases, such as Hashimoto’s thyroiditis accompanied by hypothyroidism or/and hyperthyroidism, nodule*.

**Table 5:** Thyroid hormones and autoantibodies level of oral lichen planus (OLP)/oral lichenoid lesion (OLL) and control group.

	OLP	OLL	Control	Normal reference value
Thyroid-stimulating hormone (mIU/L)	2.86 ± 2.88	2.51 ± 2.28	2.68 ± 3.51	0.3–5.0
	2.01 (0.01–19.58)	1.98 (0.006–13.87)	1.70 (0.013–26.93)	

Total thyroxine (nmol/L)	105.03 ± 26.41	108.19 ± 21.78	100.92 ± 21.20	66–154
	99.49 (62.55–194.48)	108.33 (61.78–161.90)	99.48 (42.47–153.15)	

Free thyroxine (pmol/L)	14.29 ± 3.73	14.60 ± 3.50	15.07 ± 3.50	9–25
	14.30 (1.91–33.59)	14.66 (7.9–30.35)	14.48 (9.39–29.67)	

Total triiodothyronine (nmol/L)	1.94 ± 1.55	1.79 ± 0.37	1.67 ± 0.37	1.2–2.0
	1.67 (1.01–12.16)	1.76 (0.82–2.93)	1.67 (0.79–3.00)	

Free triiodothyronine (pmol/L)	4.71 ± 0.88	4.92 ± 1.60	4.59 ± 0.85	2.1–5.4
	4.67 (2.16–8.44)	4.68 (3.30–14.92)	4.57 (2.72–7.82)	

Thyroid peroxidase antibody (IU/mL)	145.32 ± 204.17^a^	171.37 ± 298.38^a^	43.47 ± 134.94^a^	0–34
	32.92 (0.39–712.70)^a^	25.16 (0.01–1,300.00)^a^	11.20 (0.34–600.00)^a^	

	304.24 ± 458.19^b^	55.6 ± 35.74^b^	142.57 ± 316.21^b^	0–60
	73.15 (15.0–1,292.00)^b^	34.30 (28.0–107.40)^b^	30.15 (7.4–1,257.64)^b^	

Thyroglobulin (IU/mL)	203.56 ± 285.70^a^	171.96 ± 216.56^a^	173.90 ± 526.89^a^	0–115
	45.07 (10.0–1,282.70)^a^	104.73 (9.98–830.30)^a^	17.12 (9.87–2,185.00)^a^	

	146.13 ± 170.24^b^	108.24 ± 187.42^b^	67.3 ± 123.30^b^	0–60
	40.38 (3.20–500.00)^b^	27.40 (0.25–621.12)^b^	18.80 (4.69–439.23)^b^	

*The data in the top line of the table is mean value, and the bottom line is the median and range*.

*^a^Roche chemiluminescence immunoassay (CLIA)*.

*^b^Centaur CLIA*.

## Discussion

Although the comprehensive pathogenesis of OLP remains unclear, it has been reported that several systemic disorders are connected to this disorder, including metabolic syndrome, mental health disorders, and autoimmune diseases (1, 2). Of these, thyroid disease has gained an increasing interest in this field more recently. A meta-analysis has been conducted by our team and reported a close relationship between thyroid disease and OLP in many countries (9). However, the situation in Chinese patients is lacking evidence, hence the study concept presented here.

All patients in three groups come from different parts of the country, mostly north of China, such as Beijing, Hebei Province, Shandong Province, and Shanxi Provence. Since 1993, China has been popularizing the consumption of iodized salt. In 2002, the coverage rate of iodized salt in China has reached 98.72%, the iodine deficiency was largely eliminated (22). But it may probably because of the excess iodine or other reasons, there is a high prevalence of thyroid diseases in Chinese general population. In 2016, a study on the prevalence of thyroid diseases in China was conducted by Chinese Society of Endocrinology revealed that more than 40% of the population have thyroid problem (14). In this study, the prevalence of thyroid disease in OLP (72.4%) and OLL (68.8%) patients was significantly higher than the control group (49.4%), demonstrating a positive relationship between these variables.

Among all the thyroid disease, HT and hypothyroidism were most commonly identified types in OLP patients. In accordance with previous studies (23–25), we demonstrated that OLP (OR = 3.16, 95% CI = 1.87–5.33) and OLL (OR = 2.09, 95% CI = 1.18–3.70) patients showed an increased risk of HT. However, hypothyroidism showed no significant association with OLP/OLL. Interestingly, we also found a close association between thyroid nodules and OLP (OR = 2.31; 95% CI = 1.30–4.09) but not OLL (OR = 1.57; 95% CI = 0.84–2.93). These co-existing conditions have not been commonly reported in previous studies.

Hashimoto’s thyroiditis is a prevalent autoimmune endocrine diseases with a prevalence of 5–10% in the general population and is characterized by lymphocytic infiltration and fibrosis of the thyroid gland (26). This pathological characteristic seems to be very similar to the hallmark features of OLP, where there is necrosis of the basal cell layer and dense lymphocyte infiltration in the adjacent connective tissue (27). The pathogenesis of HT is far from understood. It is well accepted that the development of HT depends on an immune defect within the individual where there is genetic susceptibility in combination with causative environmental factors. Evidence indicates that environmental factors such as infections and xenobiotics play important role in the pathogenesis of HT (28). Similar as for OLP, T-cell and B-cell mediated immune responses play key roles in HT, especially the former cell type (1, 29, 30). Although the reasons for comorbidity of OLP and HT are still not well understood, it seems that OLP and HT share some common immunological triggers and pathogenic processes.

Several authors have suggested that there is a common genetic susceptibility for LP and thyroid autoimmune disease by analyzing the links between specific alleles of the *HLA* genes in these disorders (15). Hawkins et al. found that HLA-DRw9 was closely associated with some autoimmune diseases including Graves’ disease and HT in Chinese populations (31). Similarly, a recent work conducted by Lin and Sun reported the remarkably higher frequencies of occurrences of HLA-DRw9 in Chinese OLP patients (32).

Oral lichen planus and HT are both T-cell-mediated autoimmune diseases that self-antigen or exogenous agents might be the triggers of the immune response (29, 33, 34). In genetically predisposed subjects, a defensive immune reaction could be changed into an autoimmune reaction because of structural comparability between microbial antigens and human autoantigens (35). Benvenga and Guarneri have found that molecular mimicry played important role in a better understanding of AITD (36). The main infectious agents that have been linked to OLP are hepatitis C virus and *Helicobacter pylori* (37, 38), which has been reportedly linked to AITD (39–41). Some other viruses such as EB virus and cytomegalovirus have been mentioned in OLP, but these appear to be anecdotal observations (42). Further research on infection and the associated mechanisms behind this are needed.

Hypothyroidism was the most commonly reported comorbidity disease in OLP in previous studies (43, 44). However, a negative connection between OLP or OLL and hypothyroidism was observed in this study. This may be partly due to the different methodology (retrospective study versus prospective study). Previous studies have indicated that HT was the most common cause of hypothyroidism (45), but in our study, the autoantibodies level of patients classified to hypothyroidism group was normal, so the different diagnostic criteria for hypothyroidism, which might explain the different findings (16, 44) to some extent.

Thyroid nodule is a common thyroid disorder with a high prevalence in the general population. The percentages have been shown to vary depending on the mode of discovery, e.g., 2–6% (palpation), 19–35% (ultrasound), and 8–65% (autopsy data) (46–48). In the majority of cases, various stages of nodule formation and degeneration could be reflected in the thyroid nodule history, including adenoma, cyst, or colloid nodule (49). Iodine deficiency and external radiation are the most frequent factors contributing to the development of nodule. Other etiological precipitants include smoking, genetic factors, and inflammation, which are also thought to be associated with thyroid nodule (50). The association between OLP and thyroid nodule has not been commonly mentioned in previous studies, further studies are necessary to confirm this connection and fully understand the potential mechanism.

## Conclusion

In summary, this study demonstrated that OLP was closely associated with HT and thyroid nodule but not hypothyroidism. The possible mechanism behind this association is still unresolved and merits further investigation. Considering the high prevalence of thyroid disease in OLP and OLL patients, it would be useful for middle-aged women affected by OLP to be screened for thyroid diseases. As for the related mechanism of OLP and thyroid disease, it may be possible to consider the molecular simulation theory as a research hypothesis.

## Ethics Statement

This study was approved by the Peking University Institutional Review Board, China [PKUSS IRB _AF 01 _v2.0 (2014-10-01)], and all methods were performed in accordance with the relevant guidelines and regulations. Each subject agreed and provided written informed consent before the study.

## Author Contributions

HH and TZ developed the project and implementation scheme. TZ, DL, and HH recruited and collected clinical cases. DL and CL performed the statistical analysis of relevant data. TZ, CL, and DL participated in the writing and modification of the article. CL, QC, and HH critically revised the manuscript.

## Conflict of Interest Statement

The authors declare that the research was conducted in the absence of any commercial or financial relationships that could be construed as a potential conflict of interest.
